# (2*S*,3*R*)-2-[(4-Ethyl-2,3-dioxopiperazin-1-yl)carbonyl­amino]-3-hydroxy­butyric acid monohydrate

**DOI:** 10.1107/S1600536808017832

**Published:** 2008-06-19

**Authors:** Chun-xiang Ji, Yong-tao Cui, Dong-ling Yang, Cheng Guo

**Affiliations:** a Department of Applied Chemistry, College of Science, Nanjing University of Technolgy, Xinmofan Road No. 5, Nanjing 210009, People’s Republic of China; b Nanjing FroChem Tech Co. Ltd., Xinmofan Road No. 36 Nanjing, Nanjing 210009, People’s Republic of China

## Abstract

In the title compound, C_11_H_17_N_3_O_6_·H_2_O, an important building block of the medicine cefbuperazone sodium, the piperazine ring adopts a screw-boat conformation. Inter­molecular O—H⋯O and intra­molecular N—H⋯O hydrogen bonds are observed. The water mol­ecule participates as both donor and acceptor in this framework.

## Related literature

For related literature, see: Anger *et al.* (2001 ▶); Özcan *et al.* (2003 ▶); Rondu *et al.* (1997 ▶); Saikawa *et al.* (1981 ▶).
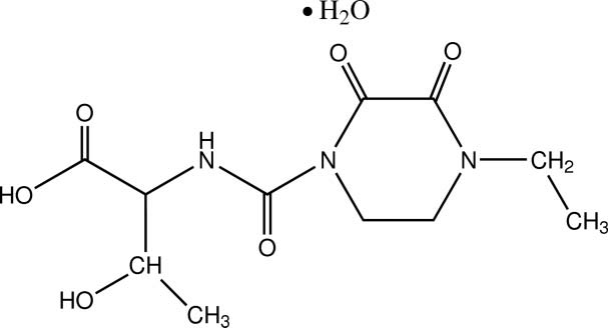


## Experimental

### 

#### Crystal data

C_11_H_17_N_3_O_6_·H_2_O
*M* *_r_* = 305.29
Orthorhombic, 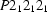

*a* = 9.4640 (19) Å
*b* = 11.389 (2) Å
*c* = 13.611 (3) Å
*V* = 1467.1 (5) Å^3^
*Z* = 4
Mo *K*α radiation
μ = 0.12 mm^−1^
*T* = 293 (2) K
0.40 × 0.30 × 0.20 mm

#### Data collection

Enraf–Nonius CAD-4 diffractometer
Absorption correction: ψ scan (North *et al.*, 1968 ▶) *T*_min_ = 0.955, *T*_max_ = 0.977
1519 measured reflections
1519 independent reflections
1287 reflections with *I* > 2σ(*I*)
3 standard reflections every 200 reflections intensity decay: <1%

#### Refinement

*R*[*F* ^2^ > 2σ(*F* ^2^)] = 0.041
*wR*(*F* ^2^) = 0.105
*S* = 1.04
1519 reflections
205 parameters
2 restraints
H atoms treated by a mixture of independent and constrained refinement
Δρ_max_ = 0.16 e Å^−3^
Δρ_min_ = −0.16 e Å^−3^

### 

Data collection: *CAD-4 Software* (Enraf–Nonius, 1989 ▶); cell refinement: *CAD-4 Software*; data reduction: *XCAD4* (Harms & Wocadlo, 1995 ▶); program(s) used to solve structure: *SHELXS97* (Sheldrick, 2008 ▶); program(s) used to refine structure: *SHELXL97* (Sheldrick, 2008 ▶); molecular graphics: *SHELXTL* (Sheldrick, 2008 ▶); software used to prepare material for publication: *PLATON* (Spek, 2003 ▶).

## Supplementary Material

Crystal structure: contains datablocks global, I. DOI: 10.1107/S1600536808017832/bh2173sup1.cif

Structure factors: contains datablocks I. DOI: 10.1107/S1600536808017832/bh2173Isup2.hkl

Additional supplementary materials:  crystallographic information; 3D view; checkCIF report

## Figures and Tables

**Table 1:** Hydrogen-bond geometry (Å, °)

*D*—H⋯*A*	*D*—H	H⋯*A*	*D*⋯*A*	*D*—H⋯*A*
N3—H3*A*⋯O2	0.86	1.99	2.647 (3)	132
O7—H7*A*⋯O2	0.85 (2)	1.98 (2)	2.817 (4)	169 (5)
O4—H4⋯O7^i^	0.75 (5)	1.85 (5)	2.593 (4)	170 (5)
O6—H6⋯O1^ii^	0.83 (4)	1.95 (4)	2.772 (3)	167 (4)
O7—H7*B*⋯O6^iii^	0.815 (19)	2.07 (3)	2.803 (4)	149 (4)

Symmetry codes: (i) 
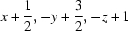
; (ii) 
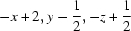
; (iii) 
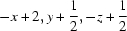
.

**Fig. 1.**
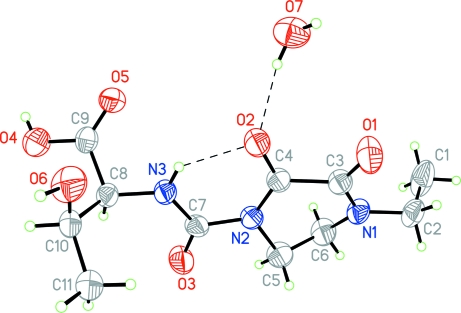
A view of the molecular structure of (I), showing displacement ellipsoids at the 30% probability level. Dashed lines indicate O—H···O and N—H···O hydrogen bonds.

**Fig. 2.**
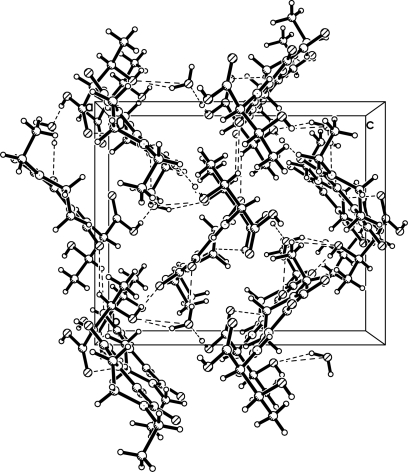
A packing diagram for (I). Hydrogen bonds are shown as dashed lines.

## supplementary crystallographic information

### Comment

Some derivatives of piperazine are important chemical materials (Saikawa *et al.*, 1981) with pharmaceutical properties (Rondu *et al.*, 1997) for example against migraine, and are calcium channel antagonist (Anger *et al.*, 2001). As part of our studies in this area, we report here the crystal structure of the title compound, (I).

The refined molecular structure of (I) is shown in Fig. 1. The title compound includes a piperzaine and a threonine moieties, and the asymmetric unit is completed by one lattice water molecule. The piperazine ring adopts a screw-boat conformation with atoms C4 and C6 displaced by 0.104 (8) and 0.596 (2) Å, respectively, from the mean plane through atoms N1, C3, N2 and C5. The dihedral angle between N1/C3/N2/C5 and N2/C7/N3/C8 planes is 4.1°.

The threonine molecular group has two chiral atoms, C8 and C10, and adopts a configuration in agreement with previous reports (*e.g*. Özcan *et al.*, 2003). The separation O6···O1 suggests an interaction between the ketone and the carboxyl group (Table 1). The water molecule is linked to the main molecule via O—H···O hydrogen bonds. These hydrogen bonds are effective in the stabilization of the crystal structure.

### Experimental

To a suspension of 2.0 g of *L*-threonine [(2*S*,3*R*)-2-amino-3-hydroxybutanoic acid] in methylene chloride (50 ml), 6.6 ml of trimethylchlorosilane were added, after which 7.1 ml of triethylamine were added dropwise at 273 K. The mixture was heated to 293 K for 2 h, and then a mixture of 4-ethyl-2,3-dioxo-1-piperazinecarbonyl chloride and triethylamine was added to the reaction mixture. After stirring for 1 h, the solvent was removed under reduced pressure. To the residue, 30 ml of water was added, and the pH was adjusted to 8 with NaHCO_3_, after which the solution was washed with 50 ml of ethyl acetate. Acetonitrile (50 ml) was added to the solution. The pH of the mixture was adjusted to 1 with HCl. The mixture was then saturated with NaCl, and the acetonitrile layer was thereafter separated. The aqueous layer was extracted with acetonitrile (3 × 50 ml), the combined acetonitrile layers were washed with saturated NaCl, and then distilled *in vacuo* to remove the solvent. The residue was recrystallized from ethanol to obtain 3.2 g of (I). Crystals of (I) suitable for X-ray diffraction were obtained by slow evaporation of an ethanol solution. ^1^H NMR (DMSO): δ 3.78–4.30 (m, 4H), 3.28–3.75 (m, 4H), 1.13 (d, 3H), 1.11 (t, 3H).

### Refinement

Hydroxyl H atoms were located in a difference map and refined freely. Water H atoms were found in a difference map and refined with a restrained geometry, O—H = 0.84 (2) Å. Other H atoms were positioned geometrically and refined using a riding model, with C—H = 0.96-0.98 Å, N—H = 0.86 Å and *U*_iso_(H) = 1.2 or 1.5 *U*_eq_ of the carrier atom. Friedel pairs were merged and the absolute configuration was assigned from starting materials.

### Crystal data

**Table:**

C_11_H_17_N_3_O_6_·H_2_O	*F*_000_ = 648
*M**_r_* = 305.29	*D*_x_ = 1.382 Mg m^−^^3^
Orthorhombic, *P*2_1_2_1_2_1_	Mo *K*α radiation λ = 0.71073 Å
Hall symbol: P 2ac 2ab	Cell parameters from 25 reflections
*a* = 9.4640 (19) Å	θ = 10–13º
*b* = 11.389 (2) Å	µ = 0.12 mm^−^^1^
*c* = 13.611 (3) Å	*T* = 293 (2) K
*V* = 1467.1 (5) Å^3^	Block, colourless
*Z* = 4	0.40 × 0.30 × 0.20 mm

### Data collection

**Table:**

Enraf–Nonius CAD-4 diffractometer	*R*_int_ = 0.0000
Radiation source: fine-focus sealed tube	θ_max_ = 25.1º
Monochromator: graphite	θ_min_ = 2.3º
*T* = 293(2) K	*h* = 0→11
ω/2θ scans	*k* = 0→13
Absorption correction: ψ scan(North *et al.*, 1968)	*l* = 0→16
*T*_min_ = 0.955, *T*_max_ = 0.977	3 standard reflections
1519 measured reflections	every 200 reflections
1519 independent reflections	intensity decay: <1%
1287 reflections with *I* > 2σ(*I*)	

### Refinement

**Table:**

Refinement on *F*^2^	Secondary atom site location: difference Fourier map
Least-squares matrix: full	Hydrogen site location: inferred from neighbouring sites
*R*[*F*^2^ > 2σ(*F*^2^)] = 0.041	H atoms treated by a mixture of independent and constrained refinement
*wR*(*F*^2^) = 0.105	*w* = 1/[σ^2^(*F*_o_^2^) + (0.0652*P*)^2^ + 0.1558*P*] where *P* = (*F*_o_^2^ + 2*F*_c_^2^)/3
*S* = 1.04	(Δ/σ)_max_ < 0.001
1519 reflections	Δρ_max_ = 0.16 e Å^−^^3^
205 parameters	Δρ_min_ = −0.16 e Å^−^^3^
2 restraints	Extinction correction: SHELXL97 (Sheldrick, 2008), Fc^*^=kFc[1+0.001xFc^2^λ^3^/sin(2θ)]^-1/4^
Primary atom site location: structure-invariant direct methods	Extinction coefficient: 0.037 (4)

### Fractional atomic coordinates and isotropic or equivalent isotropic displacement parameters (Å^2^)

**Table:**

	*x*	*y*	*z*	*U*_iso_*/*U*_eq_	
C1	0.2982 (5)	0.8688 (4)	0.3149 (3)	0.0802 (14)	
H1A	0.2231	0.9061	0.2793	0.120*	
H1B	0.3779	0.9207	0.3183	0.120*	
H1C	0.2666	0.8505	0.3802	0.120*	
C2	0.3399 (4)	0.7585 (3)	0.2637 (3)	0.0517 (9)	
H2A	0.2588	0.7066	0.2601	0.062*	
H2B	0.3683	0.7771	0.1970	0.062*	
C3	0.5900 (3)	0.7122 (3)	0.2851 (2)	0.0401 (7)	
C4	0.7064 (3)	0.6501 (3)	0.3450 (2)	0.0385 (7)	
C5	0.5156 (3)	0.5303 (3)	0.4161 (3)	0.0549 (10)	
H5A	0.4893	0.4738	0.3659	0.066*	
H5B	0.4997	0.4945	0.4798	0.066*	
C6	0.4272 (3)	0.6364 (3)	0.4065 (3)	0.0544 (9)	
H6B	0.3282	0.6145	0.4091	0.065*	
H6C	0.4461	0.6891	0.4609	0.065*	
C7	0.7632 (3)	0.4841 (3)	0.4570 (2)	0.0403 (7)	
C8	1.0033 (3)	0.4380 (3)	0.5054 (2)	0.0373 (7)	
H8A	0.9525	0.4006	0.5598	0.045*	
C9	1.1128 (3)	0.5210 (3)	0.5489 (2)	0.0390 (7)	
C10	1.0768 (3)	0.3423 (3)	0.4465 (2)	0.0425 (7)	
H10A	1.1384	0.2984	0.4913	0.051*	
C11	0.9774 (4)	0.2570 (3)	0.3990 (3)	0.0570 (9)	
H11A	1.0306	0.1992	0.3633	0.085*	
H11B	0.9161	0.2982	0.3547	0.085*	
H11C	0.9219	0.2188	0.4487	0.085*	
N1	0.4564 (2)	0.6970 (2)	0.31326 (19)	0.0416 (7)	
N2	0.6683 (3)	0.5610 (2)	0.4054 (2)	0.0397 (6)	
N3	0.9022 (2)	0.5055 (2)	0.44847 (19)	0.0383 (6)	
H3A	0.9314	0.5594	0.4091	0.046*	
O1	0.6257 (2)	0.7700 (3)	0.21398 (19)	0.0635 (8)	
O2	0.8292 (2)	0.6821 (2)	0.33274 (19)	0.0534 (7)	
O3	0.7145 (3)	0.4047 (2)	0.5047 (2)	0.0648 (8)	
O4	1.1991 (3)	0.4638 (2)	0.6080 (2)	0.0654 (8)	
H4	1.254 (6)	0.501 (4)	0.632 (3)	0.072 (15)*	
O5	1.1208 (2)	0.6236 (2)	0.53094 (18)	0.0506 (6)	
O6	1.1642 (3)	0.4009 (2)	0.3763 (2)	0.0600 (7)	
H6	1.217 (5)	0.354 (4)	0.347 (3)	0.064 (12)*	
O7	0.9118 (3)	0.9195 (3)	0.3221 (2)	0.0663 (8)	
H7A	0.894 (5)	0.847 (2)	0.332 (3)	0.080*	
H7B	0.909 (5)	0.937 (4)	0.2641 (17)	0.080*	

### Atomic displacement parameters (Å^2^)

**Table:**

	*U*^11^	*U*^22^	*U*^33^	*U*^12^	*U*^13^	*U*^23^
C1	0.071 (3)	0.088 (3)	0.081 (3)	0.038 (3)	−0.034 (3)	−0.023 (3)
C2	0.0347 (16)	0.063 (2)	0.057 (2)	0.0056 (17)	−0.0130 (16)	−0.0023 (18)
C3	0.0329 (15)	0.0434 (17)	0.0441 (17)	0.0010 (14)	0.0026 (14)	0.0032 (15)
C4	0.0277 (15)	0.0402 (16)	0.0476 (17)	−0.0004 (13)	0.0058 (14)	0.0067 (15)
C5	0.0280 (15)	0.056 (2)	0.081 (3)	−0.0076 (15)	0.0075 (17)	0.017 (2)
C6	0.0271 (15)	0.067 (2)	0.069 (2)	−0.0013 (16)	0.0102 (17)	0.016 (2)
C7	0.0325 (15)	0.0342 (15)	0.0541 (18)	−0.0024 (13)	0.0063 (15)	0.0054 (16)
C8	0.0310 (14)	0.0399 (15)	0.0410 (15)	−0.0004 (14)	−0.0004 (13)	0.0091 (15)
C9	0.0337 (15)	0.0446 (17)	0.0389 (16)	0.0053 (14)	0.0036 (14)	−0.0016 (14)
C10	0.0366 (15)	0.0394 (16)	0.0515 (17)	0.0076 (15)	−0.0094 (16)	−0.0010 (15)
C11	0.051 (2)	0.0486 (18)	0.071 (2)	0.0071 (17)	−0.016 (2)	−0.0059 (19)
N1	0.0261 (12)	0.0516 (16)	0.0471 (15)	0.0005 (11)	0.0009 (12)	0.0043 (13)
N2	0.0228 (11)	0.0387 (13)	0.0577 (15)	−0.0007 (11)	0.0055 (12)	0.0078 (13)
N3	0.0283 (12)	0.0399 (14)	0.0468 (14)	0.0006 (11)	0.0037 (12)	0.0092 (12)
O1	0.0382 (13)	0.0902 (19)	0.0622 (14)	0.0059 (13)	0.0057 (12)	0.0350 (15)
O2	0.0280 (11)	0.0555 (14)	0.0765 (16)	0.0003 (11)	0.0077 (12)	0.0252 (13)
O3	0.0400 (13)	0.0541 (15)	0.100 (2)	−0.0055 (12)	0.0030 (14)	0.0349 (15)
O4	0.0657 (18)	0.0583 (16)	0.0722 (18)	−0.0039 (15)	−0.0344 (16)	0.0042 (14)
O5	0.0444 (13)	0.0402 (12)	0.0672 (15)	−0.0013 (11)	0.0009 (12)	−0.0009 (12)
O6	0.0441 (13)	0.0687 (17)	0.0672 (16)	0.0033 (14)	0.0184 (13)	−0.0123 (14)
O7	0.0633 (16)	0.0645 (16)	0.0710 (16)	−0.0032 (15)	0.0206 (16)	−0.0012 (16)

### Geometric parameters (Å, °)

**Table:**

C1—C2	1.491 (5)	C7—N3	1.343 (4)
C1—H1A	0.9600	C7—N2	1.438 (4)
C1—H1B	0.9600	C8—N3	1.451 (4)
C1—H1C	0.9600	C8—C10	1.521 (4)
C2—N1	1.470 (4)	C8—C9	1.522 (4)
C2—H2A	0.9700	C8—H8A	0.9800
C2—H2B	0.9700	C9—O5	1.196 (4)
C3—O1	1.218 (4)	C9—O4	1.319 (4)
C3—N1	1.333 (4)	C10—O6	1.430 (4)
C3—C4	1.542 (4)	C10—C11	1.499 (5)
C4—O2	1.229 (3)	C10—H10A	0.9800
C4—N2	1.354 (4)	C11—H11A	0.9600
C5—C6	1.476 (5)	C11—H11B	0.9600
C5—N2	1.495 (4)	C11—H11C	0.9600
C5—H5A	0.9700	N3—H3A	0.8600
C5—H5B	0.9700	O4—H4	0.75 (5)
C6—N1	1.470 (4)	O6—H6	0.83 (4)
C6—H6B	0.9700	O7—H7A	0.85 (2)
C6—H6C	0.9700	O7—H7B	0.815 (19)
C7—O3	1.205 (4)		
			
C2—C1—H1A	109.5	N3—C8—C10	113.6 (2)
C2—C1—H1B	109.5	N3—C8—C9	109.1 (2)
H1A—C1—H1B	109.5	C10—C8—C9	109.8 (2)
C2—C1—H1C	109.5	N3—C8—H8A	108.1
H1A—C1—H1C	109.5	C10—C8—H8A	108.1
H1B—C1—H1C	109.5	C9—C8—H8A	108.1
N1—C2—C1	112.7 (3)	O5—C9—O4	124.6 (3)
N1—C2—H2A	109.1	O5—C9—C8	124.8 (3)
C1—C2—H2A	109.1	O4—C9—C8	110.6 (3)
N1—C2—H2B	109.1	O6—C10—C11	112.2 (3)
C1—C2—H2B	109.1	O6—C10—C8	106.4 (3)
H2A—C2—H2B	107.8	C11—C10—C8	113.9 (3)
O1—C3—N1	124.2 (3)	O6—C10—H10A	108.1
O1—C3—C4	118.0 (3)	C11—C10—H10A	108.1
N1—C3—C4	117.8 (3)	C8—C10—H10A	108.1
O2—C4—N2	123.8 (3)	C10—C11—H11A	109.5
O2—C4—C3	117.8 (3)	C10—C11—H11B	109.5
N2—C4—C3	118.3 (2)	H11A—C11—H11B	109.5
C6—C5—N2	110.3 (3)	C10—C11—H11C	109.5
C6—C5—H5A	109.6	H11A—C11—H11C	109.5
N2—C5—H5A	109.6	H11B—C11—H11C	109.5
C6—C5—H5B	109.6	C3—N1—C2	121.2 (3)
N2—C5—H5B	109.6	C3—N1—C6	119.2 (3)
H5A—C5—H5B	108.1	C2—N1—C6	118.6 (2)
N1—C6—C5	110.7 (3)	C4—N2—C7	125.9 (2)
N1—C6—H6B	109.5	C4—N2—C5	119.5 (3)
C5—C6—H6B	109.5	C7—N2—C5	114.4 (2)
N1—C6—H6C	109.5	C7—N3—C8	120.2 (3)
C5—C6—H6C	109.5	C7—N3—H3A	119.9
H6B—C6—H6C	108.1	C8—N3—H3A	119.9
O3—C7—N3	123.9 (3)	C9—O4—H4	115 (4)
O3—C7—N2	118.8 (3)	C10—O6—H6	111 (3)
N3—C7—N2	117.3 (3)	H7A—O7—H7B	113 (5)
			
O1—C3—C4—O2	16.1 (5)	C1—C2—N1—C6	72.6 (4)
N1—C3—C4—O2	−165.3 (3)	C5—C6—N1—C3	−44.8 (4)
O1—C3—C4—N2	−161.7 (3)	C5—C6—N1—C2	146.7 (3)
N1—C3—C4—N2	16.9 (4)	O2—C4—N2—C7	−6.0 (5)
N2—C5—C6—N1	55.3 (4)	C3—C4—N2—C7	171.7 (3)
N3—C8—C9—O5	−6.4 (4)	O2—C4—N2—C5	179.1 (3)
C10—C8—C9—O5	118.6 (3)	C3—C4—N2—C5	−3.2 (4)
N3—C8—C9—O4	174.5 (3)	O3—C7—N2—C4	−176.4 (3)
C10—C8—C9—O4	−60.4 (3)	N3—C7—N2—C4	3.4 (5)
N3—C8—C10—O6	67.5 (3)	O3—C7—N2—C5	−1.3 (5)
C9—C8—C10—O6	−54.9 (3)	N3—C7—N2—C5	178.5 (3)
N3—C8—C10—C11	−56.5 (4)	C6—C5—N2—C4	−32.7 (5)
C9—C8—C10—C11	−179.0 (3)	C6—C5—N2—C7	151.8 (3)
O1—C3—N1—C2	−5.0 (5)	O3—C7—N3—C8	−5.7 (5)
C4—C3—N1—C2	176.5 (3)	N2—C7—N3—C8	174.5 (2)
O1—C3—N1—C6	−173.2 (3)	C10—C8—N3—C7	101.8 (3)
C4—C3—N1—C6	8.3 (4)	C9—C8—N3—C7	−135.4 (3)
C1—C2—N1—C3	−95.7 (4)		

### Hydrogen-bond geometry (Å, °)

**Table:**

*D*—H···*A*	*D*—H	H···*A*	*D*···*A*	*D*—H···*A*
N3—H3A···O2	0.86	1.99	2.647 (3)	132
O7—H7A···O2	0.85 (2)	1.98 (2)	2.817 (4)	169 (5)
O4—H4···O7^i^	0.75 (5)	1.85 (5)	2.593 (4)	170 (5)
O6—H6···O1^ii^	0.83 (4)	1.95 (4)	2.772 (3)	167 (4)
O7—H7B···O6^iii^	0.815 (19)	2.07 (3)	2.803 (4)	149 (4)

Symmetry codes: (i) *x*+1/2, −*y*+3/2, −*z*+1; (ii) −*x*+2, *y*−1/2, −*z*+1/2; (iii) −*x*+2, *y*+1/2, −*z*+1/2.
